# Health-related quality of life of children with physical disabilities: a longitudinal study

**DOI:** 10.1186/1471-2431-14-26

**Published:** 2014-01-30

**Authors:** Mary Law, Steven Hanna, Dana Anaby, Marilyn Kertoy, Gillian King, Liqin Xu

**Affiliations:** 1 School of Rehabilitation Science, McMaster University, Hamilton, Canada; 2 Department of Clinical Epidemiology and Biostatistics, McMaster University, Hamilton, Canada; 3 School of Physical and Occupational Therapy, McGill University, Montreal, Canada; 4 School of Communication Sciences and Disorders, University of Western Ontario, London, Canada; 5 Bloorview Research Institute, Holland Bloorview Kids Rehabilitation Hospital, Toronto, Canada; 6 Canadian Partnership Against Cancer, Toronto, Canada

**Keywords:** Disability, Health-related quality of life, Longitudinal study, Environmental barriers

## Abstract

**Background:**

Outcomes of health and rehabilitation services for children and youth with disabilities increasingly include assessments of health-related quality of life (HRQoL). The purpose of this research was to 1) describe overall patterns of HRQoL, 2) examine changes in parent’s perceptions of child’s HRQoL across 18 months and 3) explore factors that predict these changes.

**Methods:**

Participants in this study included 427 parents of children (229 boys and 198 girls) with a physically-based disability between the ages of 6 to 14 years. The Child Health Questionnaire (CHQ) was administered three times, at nine month intervals. Comparisons to the CHQ normative data were analyzed at Time 1 using t-tests, and change over time was examined using linear mixed-effects models. Possible predictors were modeled: 1) child’s factors measured by the Activities Scale for Kids, Strengths and Difficulties Questionnaire, and general health measured by SF-36, 2) family characteristics measured by the Impact on Family Scale and 3) environmental barriers measured by the Craig Hospital Inventory of Environmental Factors.

**Results:**

CHQ scores of the study’s participants demonstrated significantly lower summary scores from the normative sample for both CHQ Physical and Psychosocial summary scores. On average, children did not change significantly over time for physical summary scores. There was an average increase in psychosocial health that was statistically significant, but small. However, there was evidence of heterogeneity among children. Environmental barriers, behavioral difficulties, family functioning/impact, general health and child physical functioning had negative and significant associations with physical QoL at baseline. Change in physical QoL scores over time was dependent on children’s behavioral difficulties, family functioning and environmental barriers. Environmental barriers, behavioral difficulties, family functioning/impact and general health had significant associations with psychosocial scores at baseline, but none served as predictors of change over time.

**Conclusions:**

Children with physical disabilities differ from the normative group on parent ratings of their physical and psychosocial health. While there was little average change in CHQ scores over 18 months, there is evidence of heterogeneity among children. Factors such as environmental barriers, family functioning/impact, child physical functioning and behavioral difficulties and general health significantly influence QoL scores as measured by the CHQ.

## Background

Childhood physical disability refers to intrinsic biological or acquired conditions (e.g., cerebral palsy, spina bifida, traumatic brain injury, spinal cord injury, amputation) causing impairments which result in disability and limited participation in day-to-day activities. Increasingly, outcomes of health and rehabilitation services for children and youth with disabilities include assessments of health status and well-being, known as health-related quality of life. Health-related quality of Life (HRQoL) is defined as “perceived physical and mental health over time” [1]. HRQoL measures are defined as multi-dimensional assessments of health status and well-being that include items about functional status (physical, psychological and social), well-being, and general health [2]. HRQoL assessments measure these domains from the perspective of the child/youth and/or their parents. For children with physical disabilities, health-related quality of life includes not only their functioning and participation in daily living, but also the impact of their disability on the family.

The Child Health Questionnaire (CHQ) is a self-administered or parent proxy assessment of physical, psychological and social health status of children 5–18 years of age [3]. To date, studies using the CHQ have focused primarily on cross sectional studies of children with cerebral palsy, arthritis or brain injury. In this paper, we describe parents’ perceptions of their child’s health-related quality of life (HRQoL) for 427 children with physical disabilities, the change of these perceptions across an 18-month period of time and the factors that affect amount and direction of change.

Measurement of health outcomes typically focuses on the nature and extent of functional limitations in physical, social, and psychological domains classified in ICIDH-2 [4]. The CHQ measures Health Related Quality of Life (HRQoL) across these domains [3,5]. This measure has been shown to have good reliability, validity and discriminant validity [6-8], is easy to administer [6], has normative data [4,6], and has both a parent proxy version as well as a child- completed version to allow for assessment of the perspectives of children and parents [9].

The CHQ has been used to measure the HRQoL of children with musculoskeletal or neurologically-based conditions. For musculoskeletal conditions, Selvaag et al. [10] measured the health status in a sample of 116 children with juvenile idiopathic arthritis (JIA) using the CHQ and the Childhood Health Assessment Questionnaire (CHAQ) and compared results against the results of 116 matched controls. Participants with JIA had significantly poorer physical and psychosocial health scores than healthy children. This study found that the CHQ differentiated between healthy children and children with early juvenile idiopathic arthritis and was able to measure clinical changes in children with arthritis. In another study of children with JIA, the CHQ discriminated between healthy and unhealthy children, but was not sensitive to differences between JIA subtypes [11].

Oliveira et al. [12] and Gutierrez-Suarez et al. [13] reported results from a large multinational (30 countries), cross sectional study of children (3,324 with JIA and 3,315 healthy). Results were similar to those of Selvaag et al. [10], with physical scores on the CHQ significantly lower and psychosocial scores being slightly lower than those of healthy children. Lower physical functioning scores correlated with the level of functional impairment while the intensity of pain correlated with lower psychosocial scores [12,13].

Studies with the CHQ for children with neurologically based conditions have primarily centered on children with cerebral palsy. Liptak et al. [14] examined parent reported health status of 235 children (ages 2-18) with moderate to severe cerebral palsy. Children in this study had significantly lower CHQ scores than their healthy peers in the following areas: pain, general health, physical functioning, and impact on parents. Children with more severe cerebral palsy had significantly lower scores than those with less severe cerebral palsy in each of the areas. Liptak’s study had few children with mild cerebral palsy and did not provide mean scores to compare with other studies. To address this limitation, Wake et al. [8] compared data on 80 children with CP across the severity spectrum to data of typically developing children from the same sample population base (taken 2 years prior). As with Liptak et al. [14], their results showed that parents of children with CP reported significantly lower CHQ scores for physical health and parent and family impact. The psychosocial health of the children with cerebral palsy was similar to that of and for healthy children, but the parents of children with CP reported lower psychosocial well-being and activity scores than did parents of typically developing children.

Vargus-Adams [15] surveyed 177 parents of children with CP, stratifying severity of the child’s CP using the Gross Motor Function Classification System (GMFCS), and compared the study results with the results by McCarthy et al. [5] and Liptak et al. [14]. Results indicated that scores on the CHQ were lower for children with CP (compared to healthy children) in the areas of physical functioning and parental impact, but few differences were found for psychosocial functioning between groups. In a large study of 818 children with cerebral palsy ages 8 to 12 years, Beckung et al. [16] confirmed significantly lower CHQ scores as compared to normative data. Other studies of the CHQ with children with spina bifida [17], hip dysplasia [18], admission to hospital due to injury [19,20], and chronic pain – not specified [21] report that scores on the CHQ physical functioning were lower in their samples compared with healthy children.

Changes in CHQ scores over time for children with disabilities have been examined in three studies. Vargus-Adams [22] found no significant changes over one year in 177 children (ages 3-18) with cerebral palsy. Using a longer timeframe of 2.5 years, McCullough, Parkes, Kerr & McDowell [23] examined changes in the CHQ for 184 children with cerebral palsy aged 4 to 17 years. Their findings indicate that scores for the domains of Behavior and Family Activities increased over that time period. Other domains in the measure did not change significantly. Selvaag et al. [24] found improvement in health status post intervention for 197 children with juvenile rheumatoid arthritis and juvenile spondyloarthropathy in all areas of HRQoL, except for pain, over a 3 year period. Two studies have examined changes in health-related quality of life using other measures. Janssen, Voorman, Becher, Dallmeijer & Schuengel [25] used the TNO-AZL measure with 91 children with cerebral palsy aged 8 to 14 years and found that health-related quality of life remained stable except for an increase in autonomy scores. In a study of 185 adolescents with cerebral palsy, Livingston and Rosenbaum [26] demonstrated that group scores on the Quality of Life measure and the Health Utilities Index Mark 3 (HUI3) were stable over one year. Scores on the HUI3 showed more variability, at moderate levels in speech, vision, dexterity, cognition and hearing and higher levels for pain and emotion.

Research about child-related predictors of psychosocial HRQoL indicates significant relationships for measures of child behavior [8,10,24,27], and disability level [12,14,15,17,22]. From a family and community perspective, significant predictors of psychosocial HRQoL include parent impact [15,22], family support [28], school environmental supports [17], parenting styles [29], parent’s emotional health [20] and parent’s rating of general health [12,13]. Although these findings are from cross-sectional studies, they do indicate the potential that amount and direction of change in psychosocial HRQoL over time is explained by child behavior, disability level, impact on family, family support, parents health, parenting styles and perceptions of environmental supports.

Predictors of physical HRQoL found in previous research for children with disabilities include gross motor function [14,22,23,25], disability level [8,10-13,17,22,24], parents’ ratings of general health [12] and neurologic examination results [27]. We hypothesized that children’s health and physical functioning, environmental supports, and parent’s general health would be significantly related to a measure of physical HRQoL.

In summary, there is little information regarding change in the CHQ over time with children with physical disabilities, which is important to know because patterns of changes as well as predictors of change can inform potential interventions focused on health and well-being. This study examines change in a large sample of children with musculoskeletal or neurological disabilities, thus enabling the exploration of the effect of child, family, and environmental variables on physical and psychosocial CHQ scores.

## Methods

The purpose of this research was to 1) describe overall patterns of HRQoL; 2) examine changes in parent’s perceptions of child’s health-related quality of life across an 18-month period of time; and 3) explore factors that predict these changes. The data in this paper’s analyses was gathered through a longitudinal study conducted from 2000 to 2003 focusing on the participation of school-age children with physical disabilities in activities outside of school [30,31]. Ethical approval for the study was provided by McMaster Health Sciences Research Ethics Board, which operates in compliance with the ICH Good Clinical Practice Guidelines and the Tri-Council Policy Statement: Ethical Conduct for Research Involving Humans. The sample was selected randomly from eleven regional children’s rehabilitation centers and one children’s hospital in the province of Ontario, Canada. A list of all children with physical disabilities born between October 1, 1985 and September 30, 1994 inclusive was developed. Children with primary diagnoses or conditions such as the following were included: amputation; cerebral palsy; cerebral vascular accident/stroke (vascular brain disorders); congenital anomalies; hydrocephalus; juvenile arthritis; muscular disorders (nonprogressive); neuropathy; orthopaedic conditions (e.g., scoliosis); spinal cord injury; spina bifida; and traumatic brain injury. Children with progressive disorders were excluded. Equal cohorts of boys and girls aged 6 – 8, 9 – 11, and 12 plus years old, and their families, were recruited from a potential pool of 3062 children. 509 families agreed to participate in the study. Of these 509, 40 did not meet all inclusion criteria, 28 withdrew prior to data collection and 14 were unsuitable, leaving 427 children in the study. Data collection occurred at three points in time at nine-month intervals. Informed consent was obtained from the parents of each child. Self-administered questionnaires were mailed to the family prior to a home visit by an interviewer. Measures and the interview to complete the Children’s Assessment of Participation and Enjoyment (CAPE) [32] were completed with the child and the parent most knowledgeable about his/her child.

### Participants

Participants in this study included a parent and a child respondent for each of 427 children (229 boys and 198 girls) with a physically-based disability between the ages of 6 to 14 years at baseline data collection. In Table 1, the age, sex, and health and developmental problems of the children and youth are reported. Mothers were the primary parent respondents (89%) and the majority of children lived in two-parent families (83%). Participants were predominantly of Caucasian background (81%). Fifty one percent of families reported annual incomes of less than $60,000 (compared to the median family income in the province of Ontario of $61,000) [33].

**Table 1:** Characteristics of the children in the study (N = 427)

***Characteristic***	***N = 427***	***Valid %***
Child’s sex		
Male	229	53.6
Female	198	46.4
Child’s age		
6 - 8 years	125	29.3
9 - 11 years	176	41.2
12 - 14 years	126	29.5
Child’s primary health problem		
Central nervous system	340	79.6
Musculoskeletal system	87	20.4
Child’s primary diagnostic category		
Cerebral palsy or related (CNS)	217	50.8
Spina bifida, spinal cord	52	12.2
Acquired brain injury	25	5.9
Developmental delay	12	2.8
CNS minor motor	19	4.4
CNS - other	15	3.5
Neuromuscular	20	4.7
Skeletal	54	12.7
Musculoskeletal – other	13	3.1
Missing	2	0.5

### Measures

Parent-completed measures in the study included the Child Health Questionnaire Parent Form 50 (CHQ; [3]), the Impact on Family Scale (IOF; [34]), Strengths and Difficulties Questionnaire (SDQ; [35]), Craig Hospital Inventory of Environmental Factors (CHIEF; [36]). Measures completed by the child included the Activities Scale for Kids (ASK; [37]) and the CAPE. The Short Form-36 (SF-36; [38]) was also completed and we used the general health score.

The CHQ is an assessment that examines the child’s health and quality of life from a parent or caregiver perspective, including the impact of the child’s functioning on the parent [3,39]. Domains on the CHQ include general health, family cohesion, physical functioning, change in health, limitations in schoolwork and activities with friends, bodily pain, behavior, self-esteem, mental health, limitations in family activities, and emotional or time impact on the parent. The CHQ generates 14-concept health status and well-being scores with standardized scores ranging from 0-100. Summary physical and psychosocial scores are also generated and have normative means of 50 with a standard deviation of 10. The CHQ measures HRQoL across these domains [3,5]. The CHQ has been shown to have good reliability, validity and discriminant validity [6-8], is easy to administer [6], has normative data [4,6], and has both a parent proxy version as well as a child- completed version to allow for assessment of the perspectives of children and parents [9].

Child’s behavior was measured using the SDQ [35]. This questionnaire includes 30 items (25 items related to psychological attributes of the child and 5 related to the impact of the child’s difficulties). The 25 items provide measures of Emotional Problems, Conduct Problems, Hyperactivity, Peer Problems and Prosocial Behavior. Each item is rated on 3-point scale (0 = not sure, 1 = somewhat true, 2 = certainty true) by the child’s parents. A sum score is generated for each scale, where higher scale scores and total scores mean more negative behaviors, except for the Prosocial scale where a higher score indicates more positive behavior. In addition, a total difficulties score is generated by summing all the items, with the exception of the items attributed to the Prosocial scale, resulting in 20 items and a range from 0 to 40. The SDQ has satisfactory internal consistency and test-retest reliability [40].

Physical functioning and daily task performance was measured using the ASK [37], a 30-item measure for children 5 to 15 years of age. The ASK assesses a child’s ability to perform daily tasks such as personal care, dressing, eating and drinking, and play. Scoring is based on activity independence: 0 = none of the time, 1 = once in a while, 2 = sometimes, 3 = most of the time, and 4 = all of the time. The ASK has excellent reliability (internal consistency, test-retest, inter-rater, and intra-rater reliabilities of .94 or greater) and good construct and criterion validity.

Parents’ health was measured using the SF-36 [38], an assessment of the physical, mental and social well-being of adults. The SF-36 is a generic health measure which results in eight health and well-being scores and physical and mental health summary scores. The SF-36 has excellent reliability and construct and criterion validity.

Environmental barriers were measured using the CHIEF [36], which assesses the degree to which characteristics of the physical, social, political, and institutional environment are perceived to be barriers to full participation. The CHIEF has 25 items across 5 subscales, attitudes/support, services/assistance, physical/structural, policy and work/school environmental barriers. Parents indicate the degree to which each item is a barrier to their child’s participation. The frequency scale on the CHIEF measures frequency of the barrier (“daily” to “never”) while the magnitude scale uses a dichotomous scale of “big problem” or “little problem”. Three scores are calculated for each item, a frequency score on a scale of 0-4, a magnitude score on a scale of 1-2, and a frequency-magnitude product score of overall impact which ranges from 0 to 8. The CHIEF has good test-retest and internal consistency reliability, and evidence of content, construct and discriminant validity.

Family characteristics were measured using the IOF [41]. This 24-item parent-reported scale measures financial, general, social relations and coping aspects of the family using a 4-point scale (1 = strongly agree to 4 = strongly disagree). A total score, which represents the overall impact on the family due to the child’s conditions, was calculated by the sum of 15 items as per authors’ recommendations [42] and thus ranges from 15 to 60; lower scores mean less impact on the family. The IOF is a valid and reliable scale [42].

### Analysis

The CHQ was scored following procedures described by Landgraf et al. [3]. Descriptive statistics (means and standard deviations) were calculated to examine CHQ data and the percent of the sample participants across each percentile for the CHQ subscales and summary scores at baseline or Time 1. Comparisons of the CHQ scores to the CHQ normative data from the USA were completed using t-tests, with the significance level set at p < 0.05.

A linear mixed-effects model was used to examine the change over Times 1, 2, and 3 (across 18-month study period) for the physical and psychosocial summary scores. We controlled for the child’s age since age was a predictor of lower scores in the Time 1 data. Mixed-effect models are a standard approach to the analysis of repeated-measures data and provide estimates of the average change in the sample while accounting for the correlations among repeated measurements within subjects and allowing for heterogeneity among subjects in the trajectories of change [43]. Predictors of change included: child’s factors using the total scores of the SDQ and ASK and the general health score of the SF-36; family functioning using the general score of the IOF; and environmental barriers using the CHIEF. Time was treated as categorical variable in which the third time point served as a reference level, i.e., all estimates in Time 1 or 2 were compared with Time 3. Five separate models were analyzed for the CHQ Physical score and four for the Psychosocial score to predict rates of change over time resulting in 9 models. In other words, these models tested the effect of time on CHQ scores as a function of child’s characteristics (general health, behavior difficulties, physical functioning), family features (impact on family – Physical Scale only) and environmental barriers. Models were analyzed using SAS version 9.0 and alpha was set to 0.05. 427 parents started the study and 402 completed data collection at all three time points (dropout rate of 5.9%). Parents who dropped out had lower education, lower income, were younger, and non Caucasian. The common assumption that data is missing at random was made and hence unbalanced data were allowed (this procedure is implemented within SAS). Mixed-effect models address missing data by providing numerical solutions based on all available data [44].

## Results

Mean CHQ scale and total summary scores across three data collection points over 18 months are summarized in Table 2. In Table 3, we report the mean percentile score for each CHQ subscale and summary score, as well as the percentage of children in the study who fall below the 25^th^, 50^th^ and 75^th^ percentiles for the USA normative population as published in the CHQ manual. Except for Family Cohesion, all of the percentile scores are significantly below normative values, meaning that the sample of children with disabilities had lower scores than the normative population. As shown in Table 4, the summary scales and all subscales of the CHQ, except Family Cohesion, are significantly different for this sample as compared to the CHQ normative sample.

**Table 2:** CHQ scores over the 3 measurement points

**CHQ Scales**	**Statistics**	**Time 1**	**Time 2**	**Time 3**
Family cohesion	Mean	71.87	72.12	71.85
	SD	22.02	22.37	22.34
Physical functioning	Mean	65.38	65.19	65.72
	SD	32.63	32.94	34.22
Role/social emotional/behavioral	Mean	72.39	75.32	77.23
	SD	33.05	31.91	32.15
Role/social-physical	Mean	68.62	71.25	71.28
	SD	83.33	33.72	34.64
Bodily pain	Mean	70.33	69.90	68.85
	SD	25.81	26.15	26.31
Behavior	Mean	67.71	68.91	69.10
	SD	18.29	19.14	18.77
Mental health	Mean	69.21	70.09	69.35
	SD	14.16	14.43	15.63
Self esteem	Mean	70.03	70.05	68.58
	SD	18.84	19.04	19.56
General health perceptions	Mean	60.88	61.21	62.11
	SD	21.00	20.94	21.53
Parental impact-emotional	Mean	53.38	56.02	56.61
	SD	26.13	26.01	24.97
Parental impact -time	Mean	70.14	71.53	74.66
	SD	27.68	27.09	26.33
Family activities	Mean	68.03	69.22	69.96
	SD	23.36	24.03	23.85
Physical summary score	Mean	38.32	38.68	39.05
	SD	15.90	16.54	16.71
Psychosocial summary score	Mean	44.16	45.09	45.21
	SD	11.15	11.33	11.21

**Table 3:** Percent (%) below percentile cutoff scores at Time 1 for CHQ-PF50 subscales and summary scores

**CHQ scales**	**25th %tile**	**50th %tile**	**75th %tile**	**Mean %**
Family Cohesion (FC)	12.2	45.1	45.1	45.1
Physical Functioning (PF)	76.0	76.0	76.0	76.0
Role/Social Emotional/Behavioral (REB)	54.4	54.4	54.4	54.4
Role/Social Physical (RP)	56.8	56.8	56.8	56.8
Bodily Pain and Discomfort Scale (BP)	40.4	52.6	71.4	66.7
Behavior Scale (BE)	43.2	71.1	83.8	60.3
Mental Health Scale (MH)	44.4	68.5	95.5	68.5
Self Esteem Scale (SE)	43.9	66.7	92.0	66.7
General Health Perceptions Scale (GH)	55.9	77.5	88.7	70.2
Emotional Impact on Parent Scale (PE)	72.5	81.0	95.1	81.0
Parental Impact Time Scale (PT)	61.5	73.2	73.2	61.5
Family Activities (FA)	88.3	88.3	71.1	75.8
Physical Summary Score (Phs)	76.7	84.0	91.0	79.5
Psychosocial Summary Score (PsS)	57.1	76.9	90.1	69.3

**Table 4:** CHQ results at Time 1 – Comparison to normative sample

**CHQ Scales**	**Study sample – mean (SD)**	**Normative sample – mean (SD)**	P values (ES*)	**95% confidence interval of the difference**
Family Cohesion (FC)	71.9 (22.0)	72.3 (21.6)	0.68 (0.018)	-2.5 to 1.6
Physical Functioning (PF)	65.4 (32.6)	96.1 (13.9	0.0001 (1.22)	-33.8 to -27.6
Role/Social Emotional/Behavioral (REB)	72.4 (33.0)	92.5 (18.6)	0.0001 (0.75)	-23.2 to -16.9
Role/Social Physical (RP)	68.6 (34.9)	93.6 (18.6)	0.0001 (0.89)	-28.3 to -21.6
Bodily Pain and Discomfort Scale (BP)	70.3 (25.8)	81.7 (19.0)	0.0001 (0.5)	-13.8 to -8.9
Behavior Scale (BE)	67.7 (18.3)	75.6 (16.7)	0.0001 (0.45)	-9.6 to -6.1
Mental Health Scale (MH)	69.2 (14.2)	78.5 (13.2)	0.0001 (0.67)	-10.6 to -7.9
Self Esteem Scale (SE)	70.0 (18.8)	79.8 (17.5)	0.0001 (0.53)	-11.6 to -8.0
General Health Perceptions Scale (GH)	60.9 (21.0)	73.0 (17.3)	0.0001 (0.62)	-14.1 to -10.1
Emotional Impact on Parent Scale (PE)	53.4 (26.1)	80.3 (19.1)	0.0001 (1.17)	-29.4 to -24.4
Parental Impact Time Scale (PT)	70.1 (23.3)	87.8 (19.9)	0.0001 (0.81)	-20.3 to -15.0
Family Activities (FA)	68.0 (23.3)	89.7 (18.6)	0.0001 (1.02)	-23.9 to -19.4

*ES was calculated using Cohen’s *d*; small effect = .2 to 0.49; Moderate = .50 to .79; Large ≥ .8.

Results of the linear mixed-effects model to evaluate change over time in the CHQ physical and psychosocial summary scores are reported in Tables 5, 6 and 7. The Level-1 model, presented in Table 5, tested the effect of time. On average, children did not change significantly over time for physical health scores. The average change per time in psychosocial health is small (0.6 points) and statistically significant. Table 5 also reports the standard deviations (transformed into 50% ranges) for the between child differences in Time 1 score and change over time. They give the predicted ranges within which 50% of children’s intercepts and slopes are expected to fall. For both physical and psychosocial function, children vary considerably in their predicted Time 1 scores, as well as in expected change over time. Thus, notwithstanding the lack of average change in CHQ scores, there was evidence of heterogeneity among children that was worth examining.

**Table 5:** The effect of time on rates of change in physical and psychosocial scores (level-1 model)

**Parameter**	**Physical**	**Psychosocial**
Average scores at Time 1	38.3	44.2
95% CI	[36.8, 39.8]	[43.2, 45.3]
SD of between-child differences	13.8	9.4
50% range of differences	29.0, 47.6	37.9, 50.6
Average change per time	0.3	0.6
95% CI	[–0.3, 0.9]	[0.2., 1.0]
SD of between-child differences	5.0	1.9
50% range of differences	-1.2, 1.8	-0.7, 1.9
Within-child residual SD	8.4	5.7

**Table 6:** The effect of child factors on rates of change in physical and psychosocial scores (level-2 model)

			**Physical score**		**Psychosocial score**	
	**Predictors**	**Time**	**Coefficient**	**SE**	**Coefficient**	**SE**
**Child factors**						
**SDQ**	Intercept		41.12**	1.78	59.07**	0.81
	Age		1.79	1.63	1.07	0.89
	SDQ		-0.28**	0.12	-1.18**	0.05
	Time	1	-3.53	2.04	0.26	1.25
	Time	2	-3.62**	1.71	0.48	0.95
	SDQ*time	1	0.28**	0.14	-0.08	0.08
	SDQ*time	2	0.22	0.12	-0.05	0.06
**SF-36**	Intercept		31.69**	2.75	39.14**	1.80
	Age		1.96	1.64	2.12	1.13
	SF-36		0.08**	0.03	0.08**	0.02
	Time	1	1.57	3.44	-0.38	2.4
	Time	2	1.21	3.04	0.75	2.02
	SF-36*time	1	-0.02	0.04	-0.02	0.03
	SF-36*time	2	-0.03	0.04	-0.02	0.02
**ASK**	Intercept		10.45**	2.39	43.89**	1.84
	Age		2.80	1.53	2.14	1.14
	ASK		0.36**	0.03	0.01	0.02
	Time	1	3.10	2.87	-1.31	2.05
	Time	2	-0.26	2.47	0.76	1.67
	ASK*time	1	-0.03	0.03	-0.01	0.02
	ASK*time	2	-0.001	0.03	-0.01	0.02

*p < 0.05, **p < 0.01; SE = Standard Error.

**Table 7:** The effect of family and environmental factors on rates of change in physical and psychosocial scores

			**Physical score**		**Psychosocial score**	
	**Predictors**	**Time**	**Coefficient**	**SE**	**Coefficient**	**SE**
**Family factors**						
**Impact on family**	Intercept		62.45**	2.71	++	
	Age		1.68	1.61		
	Impact		-1.20**	0.12		
	Time	1	-11.38**	3.68		
	Time	2	-9.42**	3.08		
	Impact *time	1	0.55**	0.16		
	Impact *time	2	0.42**	0.13		
**Environmental factors**						
**Overall barriers**	Intercept		44.9**	1.14	49.44**	0.75
	Age		1.26	1.58	1.64	1.06
	Barriers		-5.8**	0.6	3.83**	0.38
	Time	1	-2.01	1.4	-1.06	0.94
	Time	2	-1.48	1.2	-0.86	0.78
	Barriers *time	1	1.63*	0.8	-0.57	0.55
	Barriers *time	2	0.6	0.7	0.34	0.44

*p < 0.05, **p < 0.01; SE = Standard Error.

++ Not tested for Psychosocial score because of similarity in content for predictor variable and outcome.

The Level-2 model, testing the effect of time as a function of child, family and environmental characteristics, is presented in Tables 6 and 7. The coefficients indicated that environmental barriers had a negative and significant association with physical QoL (-5.8, p < 0.001). The significant interaction term of Barriers and Time 1 indicates that the effect of time on change in physical scores was dependent on levels of environmental barriers. That is, the relationship between the CHIEF total score and physical summary score at Time 1, compared with time 3, was significantly different. For example, when looking at the effect of environmental barriers on rates of change of CHQ physical score (Table 7), the mean initial physical score was 44.9; environmental barriers have a negative effect (beta = -5.8) on physical score. Comparing to time 3, the physical score at time 1 is significantly lower (beta = -2.01) and this change over time, i.e., 18 months, is dependent on levels of environmental barriers (beta = 0.163). In other words, environmental barriers explain the differences in physical rates (or slope) along the three data collection time points. Children with lower time 1 environmental barrier scores display greater changes in physical summary scores over time.

Behavioral difficulties had a significant and negative (-0.28, p = 0.002; -1.2) association with physical scores. The interaction effect was significant and indicated that change in physical scores over time was dependent on child’s behavioral difficulties. In other words, the relationships between behavioral difficulties and physical scores at Time 1 were different compared to Time 3. The parent’s general health and child physical functioning had a significant and positive association with physical score (β = .08, p = 0.02, β = .36, p = 0.0001 respectively) but not over time.

For change in psychosocial score (measured by CHQ), all predictors, with the exception of children physical functioning (measure by ASK), had a significant association with baseline psychosocial scores. However, none of these factors, i.e., child and environmental factors, served as predictors of psychosocial score at Times 2 or 3 and did not explain rates of change in psychosocial score over time.

## Discussion

Similar to previous studies, this study found that the health-related quality of life of children with physical disabilities was significantly less than typically developing children in the normative CHQ sample. Previous research described significant differences in the CHQ subscales of physical functioning, role functioning, parental impact and family activities, pain, and general health [8,10,12-14,23]. In the current study, there were significant differences from the normative sample across all subscales except Family Cohesion. Similar to previous studies, psychosocial scores in this sample were only slightly lower than the normative sample, a difference that is statistically significant, but may not be clinically important.

This study represents one of very few examinations of the stability of CHQ scores longitudinally. In analyzing group results, children did not change significantly over time on Physical summary scores of the CHQ. Psychosocial summary scores changed significantly over the study period of 18 months, but these changes were small and not likely to be of substantial clinical importance. Vargus-Adams [22] also found no significant changes in CHQ physical HRQoL for children with cerebral palsy over a one-year time period. In a sample of children with juvenile rheumatoid arthritis and juvenile spondyloarthropathy, Selvaag et al. [24] recorded significant improvements in health related quality of life, except for pain, over a 3-year period. These findings may be different because they are the result of an arthritis intervention study rather than an examination of naturalistic stability. The sample in this current study has predominantly central nervous system-based physical disabilities so it is not surprising that the results are more similar to the findings of Vargus-Adams [22].

These findings are similar to other studies that have explored quality-of-life measurement in children and youth with disabilities [26,45]. This result may reflect the complexity of the children's condition and their typical association with additional health or development conditions such as problems with vision, hearing, or cognition. The presence of several health or development conditions can have a greater impact on the family and is often associated with lower physical functioning. Thus, there can be considerable variability across children with similar primary health conditions. This finding may also reflect the complexity of quality-of-life and the lack of direct explanatory relationships between quality-of-life scores and child and youth functioning [46]. As well, the role of health interventions received during the study timeframe was not examined. Change in health status may be more likely in children with a less complex health condition with fewer associated neurological problems [24].

Although there was little average change in CHQ Physical summary scores, there is evidence of heterogeneity and less stability among children. The presence of heterogeneity in slopes (steepness of change over time) and intercepts (time 1 scores) was tested in level-1 model and those findings led to level-2 model analyses to identify characteristics that explained these variances. Findings indicate that heterogeneity of individual trajectories for Physical scores within this sample is explained by presence of environmental barriers, impact on family, child’s behavior and child’s physical functioning. While environmental barriers and behavior had a negative influence, physical functioning had a positive influence. In planning programs and services, these factors can be taken into account and potentially serve as entry points for intervention. For example, intervention could focus on changing environmental barriers or providing children and families with strategies to manage and change behavioral difficulties. Providing support to families in addressing issues of behavioral difficulties and environmental barriers, as well as direct support such as funding, has the potential to positively impact physical and psychosocial QoL, and should be studied further.

The sample of children with musculoskeletal-based disabilities in this study was small, comprising only 20% of the total sample and thus limiting the validity of any subgroup analyses. With so few studies examining change over time for the CHQ, it is difficult to conclude whether the measure is not responsive for children with neurologically-based disabilities or whether that population is not changing appreciably in HRQoL over time. The CHQ is a generic measure of health status so its responsiveness to change may be less than diagnostic specific measures. The advantage of this study is that linear mixed-effects models estimate both the average linear pattern of change, and the degree of heterogeneity in change among children. Thus, while scores on a group basis on the CHQ do not change significantly over time in this sample, there is less stability of scores amongst individual children. Further research regarding the ability of the CHQ to measure change over time and the best methods to analyze longitudinal health-related quality of life data is required.

The findings of this study are based on a sample from the Canadian population and must be interpreted within that context. They also reflect predominantly the perspective of mothers, who were the primary study respondents. A limitation of the study is the smaller subgroup sample size of children with musculoskeletal conditions. Further research can compare HRQoL between children with musculoskeletal versus central nervous system-based disabilities. The CHQ-PF50 was found to have low reliability for ambulant children with cerebral palsy in the domains of Behavior and General Health [23]. Although the current sample did not include a majority of children with ambulant cerebral palsy, this is a potential limitation to the study.

## Conclusions

The findings of this study support earlier research that there are significantly lower scores in the physical domain of health-related quality-of-life than the psychosocial domain for children with physical disabilities. For physical health-related quality-of-life, the findings confirm the relationship between a child's general health, physical functioning and the physical area of quality-of-life. New knowledge generated from this research indicates that the perceived impact of a disability on the family and perceived environmental supports and barriers also predict physical health-related quality-of-life. From a clinical service and policy perspective, knowledge of this relationship points out the potential importance of providing support to parents and addressing environmental barriers.

## Competing interests

The authors declare that they have no competing interests.

## Authors’ contributions

ML, GK, SH and MK participated in the design and implementation of the study. ML, DA and GK examined the literature to develop predictive hypotheses for the study. SH and DA supervised the statistical analyses which were carried out by LX. All authors helped draft the manuscript, read and approved the final manuscript.

## Pre-publication history

The pre-publication history for this paper can be accessed here:

http://www.biomedcentral.com/1471-2431/14/26/prepub
